# Efficacy of Ventilation, HEPA Air Cleaners, Universal Masking, and Physical Distancing for Reducing Exposure to Simulated Exhaled Aerosols in a Meeting Room

**DOI:** 10.3390/v13122536

**Published:** 2021-12-17

**Authors:** Jayme P. Coyle, Raymond C. Derk, William G. Lindsley, Francoise M. Blachere, Theresa Boots, Angela R. Lemons, Stephen B. Martin, Kenneth R. Mead, Steven A. Fotta, Jeffrey S. Reynolds, Walter G. McKinney, Erik W. Sinsel, Donald H. Beezhold, John D. Noti

**Affiliations:** 1 Health Effects Laboratory Division, National Institute for Occupational Safety and Health, Centers for Disease Control and Prevention, Morgantown, WV 26505, USA; nti2@cdc.gov (J.P.C.); rhd8@cdc.gov (R.C.D.); czv3@cdc.gov (F.M.B.); oph6@cdc.gov (T.B.); wrw0@cdc.gov (A.R.L.); jsr0@cdc.gov (J.S.R.); wdm9@cdc.gov (W.G.M.); eur2@cdc.gov (E.W.S.); zec1@cdc.gov (D.H.B.); ivr2@cdc.gov (J.D.N.); 2 Respiratory Health Division, National Institute for Occupational Safety and Health, Centers for Disease Control and Prevention, Morgantown, WV 26505, USA; stm9@cdc.gov; 3 Division of Field Studies and Engineering, National Institute for Occupational Safety and Health, Centers for Disease Control and Prevention, Cincinnati, OH 45226, USA; kcm3@cdc.gov; 4 Facilities Management Office, National Institute for Occupational Safety and Health, Centers for Disease Control and Prevention, Morgantown, WV 26505, USA; sff4@cdc.gov

**Keywords:** indoor exposure, ventilation, universal masking, physical distancing, HEPA air cleaner, SARS-CoV-2

## Abstract

There is strong evidence associating the indoor environment with transmission of SARS-CoV-2, the virus that causes COVID-19. SARS-CoV-2 can spread by exposure to droplets and very fine aerosol particles from respiratory fluids that are released by infected persons. Layered mitigation strategies, including but not limited to maintaining physical distancing, adequate ventilation, universal masking, avoiding overcrowding, and vaccination, have shown to be effective in reducing the spread of SARS-CoV-2 within the indoor environment. Here, we examine the effect of mitigation strategies on reducing the risk of exposure to simulated respiratory aerosol particles within a classroom-style meeting room. To quantify exposure of uninfected individuals (Recipients), surrogate respiratory aerosol particles were generated by a breathing simulator with a headform (Source) that mimicked breath exhalations. Recipients, represented by three breathing simulators with manikin headforms, were placed in a meeting room and affixed with optical particle counters to measure 0.3–3 µm aerosol particles. Universal masking of all breathing simulators with a 3-ply cotton mask reduced aerosol exposure by 50% or more compared to scenarios with simulators unmasked. While evaluating the effect of Source placement, Recipients had the highest exposure at 0.9 m in a face-to-face orientation. Ventilation reduced exposure by approximately 5% per unit increase in air change per hour (ACH), irrespective of whether increases in ACH were by the HVAC system or portable HEPA air cleaners. The results demonstrate that mitigation strategies, such as universal masking and increasing ventilation, reduce personal exposure to respiratory aerosols within a meeting room. While universal masking remains a key component of a layered mitigation strategy of exposure reduction, increasing ventilation via system HVAC or portable HEPA air cleaners further reduces exposure.

## 1. Introduction

SARS-CoV-2, the virus that causes COVID-19, can spread between humans by respiratory fluid aerosols and droplets released during exhalatory events, such as breathing, coughing, talking, singing, and sneezing [1,2,3]. Aerosols have been defined as airborne particles <5 µm in diameter and droplets as ≥5 µm in diameter [4]; however, more recent work based on a better understanding of aerosol physics defines aerosols as <100 µm with droplets being >100 µm [5], which is the definition used in this investigation. These respiratory aerosols and droplets, the smallest of which may remain suspended in the air for several minutes to several hours, can disperse throughout the indoor environment [6]. During that time, potentially infectious aerosols suspended in the indoor environment may translate to exposure and possible transmission. The risk of infection increases with the intensity and duration of exposure, as well as the concentration of active virus-laden respirable aerosols [7]. Since the critical dose threshold for SARS-CoV-2 infection is unknown, one goal of exposure mitigation is to reduce intensity, duration, or both as much as feasible. To reduce the risk of SARS-CoV-2 transmission in indoor spaces, the U.S. Centers for Disease Control and Prevention (CDC) recommends a combination of measures including universal mask wearing, increased room ventilation and filtration, and physical distancing [8].

Masking reduces the expulsion of respiratory aerosols and droplets from the point of generation [9,10,11]. Donning a mask can reduce the release of respiratory aerosols and droplets by 85% or more, depending on the mask and exhalatory event [12,13,14]. Donning a mask also may reduce the exposure of an uninfected wearer to aerosols from a potentially infectious source [15,16]. The combination of source masking and recipient masking for all individuals (called “universal masking”) can reduce exposure significantly more than when masks are just worn by a source or recipient. Modifications aimed to improve face mask fit can improve their performance. Double masking, such as by wearing a 3-ply cotton cloth mask over a medical mask, can provide up to a 96% reduction in aerosol exposure compared to no masking [14], thus providing significant protection against the transmission of SARS-CoV-2. Reduction in infectious transmission of SARS-CoV-2 by universal masking has been examined using computational modeling [17]. Further, population-level community infection studies lend support to universal masking as a manner to reduce infectious disease transmission [18,19].

Ventilation in the indoor environment may also play a role in infectious disease transmission. Since the average individual spends greater than 90% of the day within an indoor environment [20], investigations have focused on indoor transmission. An examination of 318 case clusters in China showed that all but one likely occurred with indoor settings while one cluster of two cases was attributed to interaction outdoors [21]. More granular case cluster investigations noted an increasing association between poorly ventilated indoor environments and infectious transmission [22,23]. Therefore, increasing the ventilation in a room or building provides a method for reducing exposure to potentially infectious aerosols and droplets.

A sample of classrooms among 37 U.S. schools were used to estimate effective air exchange rates typically below 2.0 air changes/hour (ACH) during daytime occupation, depending on the heating, ventilation, and air conditioning (HVAC) system configuration and ACH estimation method [24]. Since existing HVAC systems within a given structure typically have a limited capacity for increased air exchange rates, the CDC and American Society of Heating, Refrigerating and Air-Conditioning Engineers (ASHRAE) have recommended the use of portable high efficiency particulate air (HEPA) cleaning units to augment the clearance of potentially infectious aerosols [25,26]. HEPA air cleaners can provide a rapid, relatively inexpensive solution to increasing ACH as opposed to the option of renovating an existing HVAC system. Indeed, portable HEPA air-cleaning units have been shown effective in significantly reducing particle concentrations, while also contributing to changes in aerosol dispersal and distribution due to changes in room airflow patterns [27]. Further, it must be noted that the efficacy of portable HEPA air cleaners can be influenced by the airflow setting, placement position, and room occupancy [28].

Physical distancing is a behavioral mitigation strategy with the focus of limiting aerosol and droplet transmission between individuals by creating physical space between a transmission source and recipient. The physical distancing recommendation varies by jurisdiction, i.e., 1 to 2 m [29,30]. The concept of physical distancing is partially based on the assumption that droplets of respiratory fluids follow a ballistic trajectory from the point of generation and fall out of the exposure path within 2 m [31,32]. As the particle diameter decreases below 100 µm, however, a gradual transition occurs where the settling velocity rapidly decreases and the particles remain airborne longer [33]. Air currents such as plumes of warm air rising from the body can lift these particles and extend the time for which they stay in the air. Aerosols produced by coughing may travel up to 8 m [34], well beyond the often-cited short-range distance limit of 2 m. Depending upon environmental conditions, respiratory aerosols and droplets can evaporate within seconds [35], leading to increases in the airborne residency time. Further, room airflow patterns and physical orientation, e.g., face-to-face, side-by-side, or front-to-back, between two individuals can also influence exposure, thus complicating the relationship between physical distancing and exposure reduction. This improved understanding of the behavior of aerosols and droplets during exhalation plume dispersion over distance [36,37] and the volumetric dilution that occurs during mixing by ventilation and air currents has prompted a re-examination of the mechanisms by which physical distancing and exposure reduction are associated [38].

The purpose of the current investigation is to examine the efficacy of a matrix of physical distance, increasing ventilation (HVAC and HEPA purifiers), and universal masking to reduce aerosol exposure within a classroom-style meeting room. The results of the current investigation will add to the understanding of the influence of each mitigation strategy, as well as the combination thereof, to potentially reduce exposure to infectious respiratory aerosols such as those which transmit SARS-CoV-2.

## 2. Materials and Methods

### 2.1. Simulators, Masking, and Aerosol Measurement

**Figure 1:**
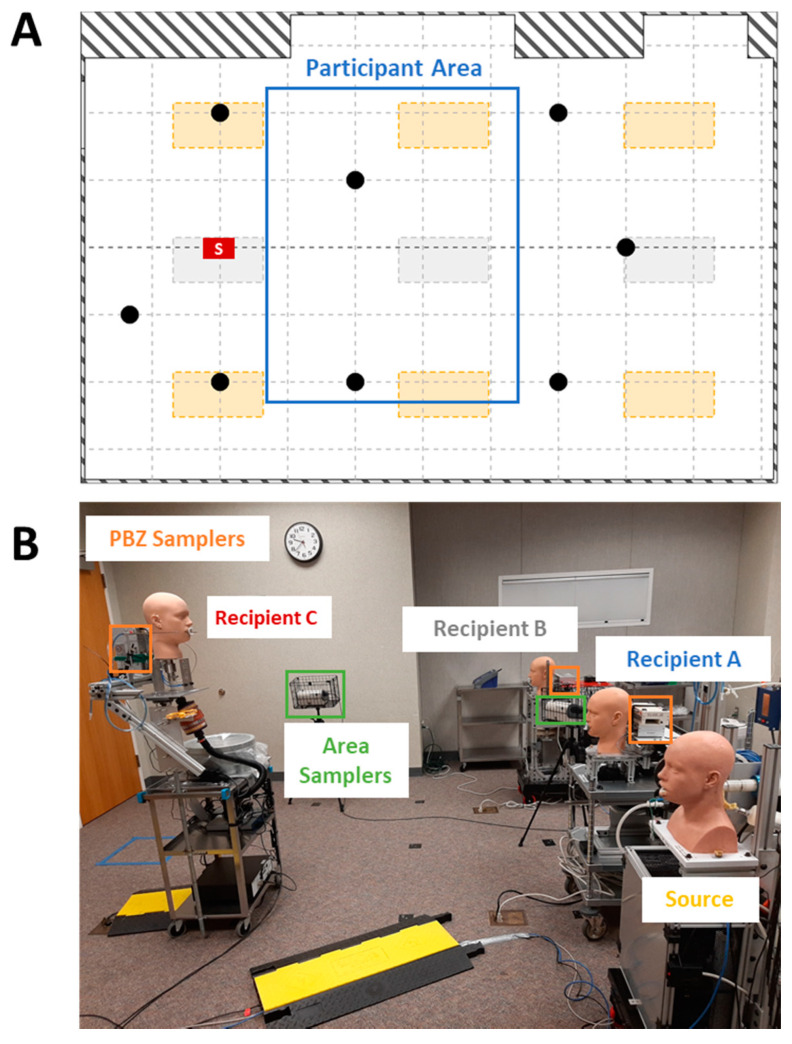
Meeting room. (**A**) Layout of the 6.6 m by 9.1 m meeting room with positions of breathing simulators and optical particle counters relative to the dimensions of the room. The “S” position indicates the position from which an individual addresses the participants. The blue box indicates the participant area in which the breathing simulators were located among all orientations used in this study. Each gridline is evenly spaced 0.9 m. Black dots denote locations of the Model 3330 optical particle counters. The orange rectangles denote the location of the HVAC system supply slot diffusers. Gray rectangles denote the location of the HVAC system return air diffusers. (**B**) Recipient (A and B) and Source simulators positioned 1.8 m from Recipient C which was positioned as the speaker. The personal breathing zone (PBZ) samplers are demarcated in orange and area samplers in green.

**Figure 2:**
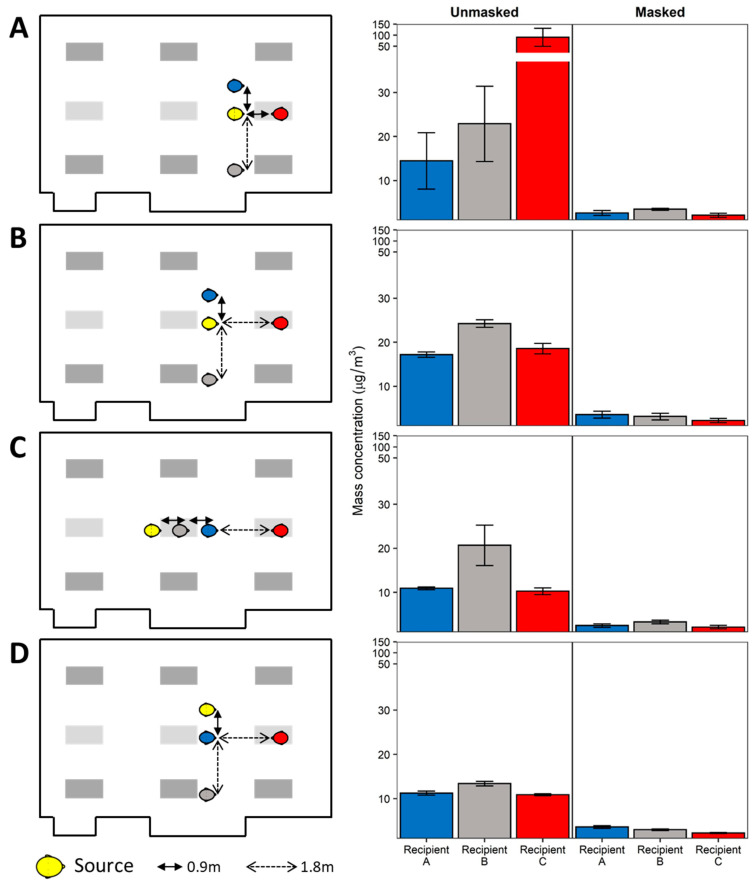
Participant aerosol source scenarios. The aerosol Source and Recipient breathing simulators were positioned in four placement configurations and the HVAC system ventilation was set to 4 ACH. The Recipient and Source simulators were positioned in (**A**) front row, (**B**) second row, (**C**) linear, and (**D**) second row source leftmost orientations. Mean mass concentrations measured at the Recipient breathing simulators for unmasked and universal masking experiments are shown. Data are presented as the mean of four independent experiments. Error bars represent one standard deviation.

**Figure 3:**
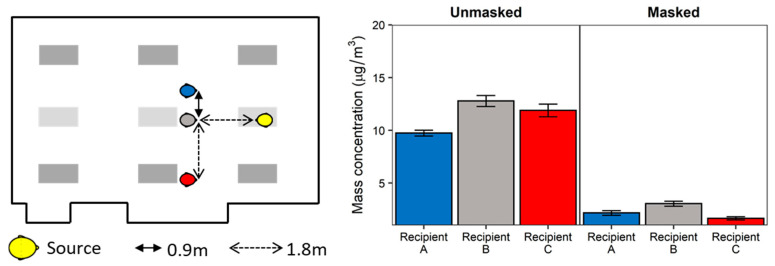
Speaker Source configuration. The Source and Recipients were positioned in a single scenario as shown in the diagram. The HVAC system ventilation was set to 4 ACH. Mean mass concentrations were measured at the Recipient breathing simulator for unmasked and universal masking experiments. Data are presented as the mean of four independent experiments. Error bars represent one standard deviation.

To better understand the effects of physical distancing, masking, and ventilation, experimentation was conducted simulating a classroom-style meeting room setup with a speaker being positioned at the front of the room and multiple participants (Figure 1). Each experiment simulates a scenario of one respiratory aerosol simulator (Source) as an infected individual and three noninfected breathing simulators (Recipients). The distance from floor to mouth of the breathing simulator at the speaker’s position was 152 cm to simulate an adult standing, while the mouths of the participant breathing simulators were 76 cm from the floor. Four different scenarios of the simulators were examined and are shown in Figure 2 and Figure 3 for reference. For an additional position scenario, Recipient C was repositioned into the audience and the Source simulator was placed in the speaker position.

Recipient C was purchased from Warwick Technologies Ltd. (Warwick, UK) while the remaining simulators (Source and Recipients A and B) were custom-built. The Warwick breathing simulator has been described in detail elsewhere [39]. The Source simulator headform was purchased from Hanson Robotics (Plano, TX, USA) while Recipients A and B simulator headforms were from Crawley Creatures Ltd. (Model: Respirator Testing Head Form 1; Buckingham, UK); all simulator headforms were composed of a shell of elastomer to mimic human skin.

The Recipient C used a sinusoidal breathing waveform with a respiration rate of 21.5 breaths/min and a ventilation rate of 27 L/min, which is approximately the average of the International Organization for Standards (ISO) standards for males and females engaged in moderate work [40]. Participant A and Participant B used an elastomeric bellows controlled by a computer-enabled linear motor and breathed with a constant sinusoidal waveform calibrated to 12 breaths/min at a tidal volume of 1.25 L/breath, resulting in a breathing ventilation rate of 15 L/min. The breathing parameters for these two simulators correspond to females performing light work [40]. The aerosol exhaled by the source simulator has a mass median aerodynamic diameter (MMAD) of 1.3 µm with a geometric standard deviation (GSD) of 2.3 [13]. 

The face masks were 3-ply cotton cloth masks with ear loops (Defender; HanesBrands Inc.; Winston-Salem, NC, USA). Experiments were conducted with all simulators either unmasked or masked (universal masking). To assess mask fit, fit factors were determined using the PortaCount Pro+ (Model 8038; TSI Corporation; Shoreview, MN) in N99 mode as per manufacturer’s instructions.

To determine the aerosol particle exposure of each Recipient, the concentrations of particles between 0.3 and 3 µm were measured at the mouth of each Recipient using optical particle counters (OPCs) (Model 1.108; Grimm Technologies, Inc.; Douglasville, GA, USA). When the breathing simulators were wearing face masks, the particle counters affixed to the Recipients collected aerosol samples from inside the masks (i.e., the particle counter measured the concentration of the aerosol being inhaled by the Recipient).

### 2.2. Meeting Room Layout and Ventilation

The meeting room used for experimentation had nominal dimensions of 6.6 m wide by 9.1 m long (with small cut-out areas that subtracted from the floor area) and a height approximately 3 m from floor to ceiling. Considering the cut-out areas, the floor surface area and room volume were 54.3 m^2^ and 164.0 m^3^, respectively. Airflow to the meeting room originated from a building air handler unit (AHU) with an economizer and variable frequency drive. The AHU was set to deliver 55 °F supply air to variable-air-volume (VAV) boxes that provide supply air to the meeting room and many other rooms on three floors of the building. The supply air first passed through a set of prefilters (HC MERV 10 Pleated Air Filter; Filtration Group; Mesa, AZ, USA) and then passed through a MERV 13 V-Bank filter (DuraMAX 4v; Koch Filter Corporation; Louisville, KY, USA). For this testing, the controls to the VAV boxes were overridden to ensure a constant airflow rate throughout each test. The air supply entered the meeting room through six 0.6 m × 1.2 m fluorescent light slot diffusers, all controlled by the same VAV box. The slot diffusers were evenly distributed with three diffusers along each longitudinal wall. The return air entered into a ceiling plenum through three 0.6 m × 1.2 m fluorescent light diffusers located through the midline of the room (Figure 1). The meeting room used for testing received less than 4% of the total amount of supply air provided by the AHU. Given this, any effects from air recirculation, as opposed to a 100% single-pass airflow delivery, were negligible to our test methodology and ignored for the purpose of this work.

The HVAC system clearance rates were determined using three methods: an HVAC measurement/calculation method based on the measured total HVAC clean air supply rate (room was positive pressure so supply air was measured instead of the return air), a tracer gas decay method using sulfur hexafluoride tracer gas, and an aerosol decay method using potassium chloride (KCl) aerosols. 

For the HVAC measurement/calculation method, the HVAC supply rates at each of the six supply inlets were measured using an Alnor Balometer with a 0.6 m × 1.2 m Capture Hood (Model EBT731, TSI Corporation) and summed. This air supply rate was divided by the volume of the meeting room to estimate the air volume displacement rate, expressed as ACH.

As an alternative to the HVAC measurement/calculation ACH, an effective ACH (ACH_eff_) was measured using tracer gas decay measurements collected from within the room’s occupiable space. Four Innova Photoacoustic Infrared Spectroscopy Analyzer models, 1412, 1412i (2×), and 1512 (California Analytical Instruments Inc.; Orange, CA, USA), were placed throughout the meeting room. All units were equipped with sulfur hexafluoride (SF_6_)-specific optical filters. The SF_6_ tracer gas (99.8% purity; Scott Specialty Gases Inc.; Plumsteadville, PA, USA) was released into the meeting room and allowed to mix to a target concentration of approximately 10 ppm. Mixing was aided by one 12′′ diameter desktop vane axial fan and one larger 24′′ diameter pedestal-base vane axial fan, in addition to the HVAC system ventilation. The fans were then turned off, and SF_6_ concentrations were continuously measured at a sample frequency of approximately 1.2–1.5 samples/min (each instrument was slightly different) for at least 30 min to document the decay rate at each of the four sample positions. After each test, the four analyzers were randomly shuffled among the four analyzer locations to reduce the potential impact of any instrument bias. Since the tracer gas could be recirculated by the AHU (although it was highly diluted), a concentration of 50 ppb SF_6_ was considered an acceptable background concentration prior to initiating the next test. The SF6 concentration decays for the four analyzers were individually plotted as a simple exponential decay using Microsoft Excel (Redmond, WA, USA). The slope of each decay curve represented the air exchange rate for each instrument location. The tracer gas-based overall room ACH_eff_ was determined by averaging the four localized air exchange rates.

Similar to the tracer gas decay method, the aerosol concentration decay method was also used to determine an ACH_eff_ rate. The meeting room was dosed with aerosols from a 14% KCl solution atomized using a 3-jet Collison jet atomizer for 20 min; a 24′′ diameter pedestal-base vane axial fan provided mixing prior to aerosol measurement. Aerosol concentrations were quantitated for a minimum of 20 min during the aerosol decay phase using eight symmetrically spaced optical particle counters (Model 3330, TSI Corp.) throughout the room—each sampling at 1 s intervals. Particle counts for the three measurement size bins, 0.3–0.4 µm, 0.4–0.5 µm, and 0.5–0.65 µm, were aggregated together for each instrument and plotted as a simple exponential decay using the R statistical environment v. 4.0.2 (R Project for Statistical Computing; Vienna, Austria). The slope of each decay curve represented the air exchange rate for each OPC location and was averaged among all OPCs for the particle-based overall room ACH_eff_.

### 2.3. HEPA Air Cleaners

The HEPA air cleaners were selected using three criteria: (1) units were listed on the Association of Home Appliance Manufacturers (AHAM) certified room air cleaners list; (2) units were readily available to the public from local suppliers or available by purchase online; and (3) units were selected based on the size of the room (including an adjustment for ceiling height above 8′) using the clean air delivery rate (CADR) for smoke particles. To augment the HVAC system, two portable HEPA air cleaners (Honeywell 50250-S; Kaz Inc.; Memphis, TN, USA) were placed in various positions throughout the meeting room. This style of unit has a 360° air intake around the sides, draws air through an activated carbon prefilter and then a HEPA filter, and expels air through the top at 360°. Each selected HEPA air cleaner was CADR-smoke rated to provide 0.12 m^3^/s (250 ft^3^/min) of air filtration which corresponded to an equivalent ACH (ACH_equiv_) rate of 2.6. The number of HEPA air cleaners determined for examination was based on meeting or exceeding the clean air delivery rate “2/3 Rule” as recommended by the Environmental Protection Agency [25,41]. For the size of the meeting room, the minimum recommended total CADR of 0.229 m^3^/s (485 ft^3^/min) was exceeded with two of the selected HEPA air cleaners, which collectively provided a CADR of 0.24 m^3^/s (500 ft^3^/min) on the maximal airflow setting. Experiments using a single HEPA air cleaner were below the recommended CADR but represented scenarios of units not meeting the “2/3 Rule” recommendation.

One or two HEPA air cleaners were placed on the floor of the meeting room, except for the raised configuration in which the HEPAs were placed on 0.8 m high tables. For all HEPA air cleaner augmentation studies, the HVAC system supply was fixed at 2 ACH which is a ventilation rate representative of office buildings [42]. The ACH_equiv_ rates of the HEPA augmentation studies were conducted using the KCl aerosol decay rates as described above, yielding a total ACH (ACH_tot_) which reflected the contributions of both the HVAC ACH and the HEPA ACH_equiv_ for each configuration. A Real Time Octave Band Analyzer (Model 407790; Extech Instrument; Nashua, MA, USA) was used to assess background noise levels during HEPA operation. Noise measurements were taken at the location of the eight area samplers between aerosol testing since the breathing simulators and aerosol source generate significant levels of noise.

### 2.4. Test Procedure

The HVAC system and HEPA air cleaners were run at the test flowrates for approximately 10 min prior to initialization of the Recipient breathing simulators and their personal breathing zone OPC sampling (Grimm samplers). The area samplers (Model 3330 TSI) were initialized concomitantly with the personal breathing zone samplers (colocated at each Recipient breathing simulator location). Background particle concentrations for the three minutes preceding the start of the source aerosol generation were used to determine background aerosol concentrations. At test time zero, the Source breathing simulator was activated and executed the aerosol generation cycle. For these experiments, a 14% *w/v* solution of KCl in distilled water was nebulized on a 1 min cycle comprising 20 s of constant nebulization via a single jet Collison jet atomizer (BGI Sciences), followed by 40 s without nebulization; this cycle continued for the 60 min duration of the test. The aerosol was passed into the elastomer bellows of the Source that breathed continuously at a rate of 15 L/min and exhaled through the mouth simulator into the meeting room. At the end of each test, the meeting room doors were opened, the HVAC system was set to 8 ACH, and the HEPA air cleaners were turned on, to reduce particle concentrations back to room baseline prior to starting the next test. Each experimental condition was repeated four times. Ambient conditions were measured using a temperature and relative humidity probe and data logger (Vaisala Oyj; Vantaa, Finland).

### 2.5. Data Processing and Statistical Analysis

Size-binned particle count data and elapsed time reported by each Grimm and Model 3330 OPC were imported and processed using the R statistical environment. Bin-specific particle counts for the 180 s observed at each OPC preceding the start of aerosol generation were used to estimate the background aerosol concentration, which was then subtracted from the subsequent OPC particle counts, prior to conversion to OPC-specific bin aggregation. The mass of the aerosol in each size bin per m^3^ of air (mass concentration) was calculated by multiplying the particle count by the volume of an individual particle with the mean diameter of the size bin (assuming the particles were spherical) and by 1.984 g/cm^3^ (the density of KCl). Note that this conversion from particle counts to particle mass is commonly used but is an approximation. For each OPC, the background-corrected, total aerosol mass concentration was averaged over 60 min to determine the mean aerosol mass concentration (mean aerosol exposure) to which each Recipient was exposed. For regression analysis, mean mass concentration was regressed against the experimental variables of physical distance from the source; relative positional orientation from the source, i.e., face-to-face, side-by-side, or front-to-back; ACH_tot_; and masking status, i.e., no masks or universal masking, using a multivariate linear model robust against heavy-tailed residual distributions [43] in R using the “*heavy*” package after outlier detection and removal. Post hoc two-sample significance tests were conducted using the Wilcoxon rank-sum test in R. Point estimates presented in the text, figures, and tables are the arithmetic mean ± 1 standard deviation of the mean aerosol exposure in units of µg/m^3^. Statistical significance was set at *p* < 0.05.

Area samples measured from the Model 3330 OPCs provided the data to generate mean mass concentration spatial interpolated overlays by inverse distance weight modeling using the “*gstat*” package in R. First, a grid of evenly spaced points throughout the exposure plane was constructed, over which predicted OPC mean mass concentrations were fitted from the observed data. The mean mass concentration range was fixed between 2.9 and ≥28 µg/m^3^. Values greater than 28 µg/m^3^ were colorized analogous to the maximum value in order to increase resolution at the lower concentrations.

## 3. Results

### 3.1. Meeting Room Conditions and Mask Fit

Across all experiments, the temperature of the meeting room was 22.6 °C ± 1.1 °C with a relative humidity of 36.7% ± 9.6%. Using the previously described HVAC measurement/calculation method, the HVAC system was determined to provide three air exchange rates to the meeting room, depending on the HVAC fan setpoint: 2.09 ACH (0.095 m^3^/second, denoted as 2 ACH), 4.07 ACH (0.185 m^3^/second, denoted as 4 ACH), and 6.08 ACH (0.277 m^3^/second, denoted as 6 ACH). At the same three HVAC operational setpoints, the average effective ACH rates (ACH_eff_) determined by the particle decay were 1.89 ACH, 3.45 ACH, and 5.03 ACH, respectively. The average ACH_eff_ results determined from the tracer gas decay measurements fell between the particle decay ACH_eff_ determination and the HVAC measurement/calculation ACH determinations (Supplemental Table S1).

When operating the HVAC system ventilation at 2 ACH, inclusion of HEPA air cleaners increased the particle decay rate by approximately 2.4–2.6 ACH per HEPA air cleaner, with slight variation in ACH_tot_ observed across all HEPA placements (Table 1). For example, the ACH_tot_ rate when a single HEPA air cleaner was placed at the front of the room, i.e., close to the aerosol source, was 0.25 ACH higher than when a single HEPA air cleaner was placed at the rear of the room. Two HEPA air cleaners further increased the total particle clearance compared to one unit, with the highest ACH_tot_ (7.14 ACH) occurring when the HEPA air cleaners were at the front and back of the room. Placement of both HEPA air cleaners at the opposing sides and within the center of the room increased the overall ACH_tot_ to 7.03 and 6.94 ACH, respectively, while raising the HEPA air cleaners onto a 0.8 m table at the opposing sides of the meeting room resulted in the lowest ACH_tot_ for two air cleaners of 6.74.

Since the procedure for mask fit testing was consistent among all experimental conditions, the results for all fit tests were summarized across all fit tests performed. Fit factor for the Source was 1.9 ± 0.3 (mean ± SD). Fit factors for Recipients A, B, and C were 3.5 ± 1.2, 3.8 ± 0.7, and 4.5 ± 1.8, respectively.

### 3.2. Source Placement

The effect of physical distance, breathing simulator orientation (i.e., face-to-face vs. side-by-side), and universal masking on exposure reduction with the HVAC system ventilation at 4 ACH are presented in Figure 2 and Figure 3. When the aerosol Source breathing simulator was positioned 0.9 m in a face-to-face orientation with the Recipient in the speaker position and all breathing simulators were unmasked, the OPC at the mouth of the Recipient in the speaker position registered the highest mean mass concentration among all scenarios (Figure 2A). In the unmasked condition, increasing the distance between the Source and the speaker (Recipient C) to 1.8 m from 0.9 m significantly reduced exposure by 79.4% (*p* = 0.029). Beyond 1.8 m, no perceptible exposure differences were noted (Figure 2B–D). When the positions of the remaining Recipients relative to the Source changed, no consistent overall pattern was noted with the exception that Recipient B tended to receive higher exposures compared to Recipient A, despite being positioned further away from the Source. When adjusting for physical distance and orientation, universal masking significantly reduced exposure by 81.6% (SD 5.9%, *p* < 0.001) compared to unmasked scenarios (Supplemental Table S2). Likewise, increasing physical distance reduced exposure by 7.9% per meter (SD 3.9%, *p* = 0.047), while orientation was not a significant predictor of overall exposure.

Placing the Source in the speaker’s position resulted in similar exposure reduction as that observed when Source placement was among the Recipients, though orientation was not investigated since all breathing simulators were face-to-face with the Source breathing simulator (Figure 3). Adjusting for physical distancing, the effect of masking remained the strongest predictor of exposure reduction (72.0% ± 1.4%, *p* < 0.001). Physical distancing with the Source as the speaker reduced exposure to a similar extent as the Recipient source scenarios (9.5% ± 1.9%, *p* < 0.001, Supplemental Table S3).

### 3.3. Ventilation

**Figure 4:**
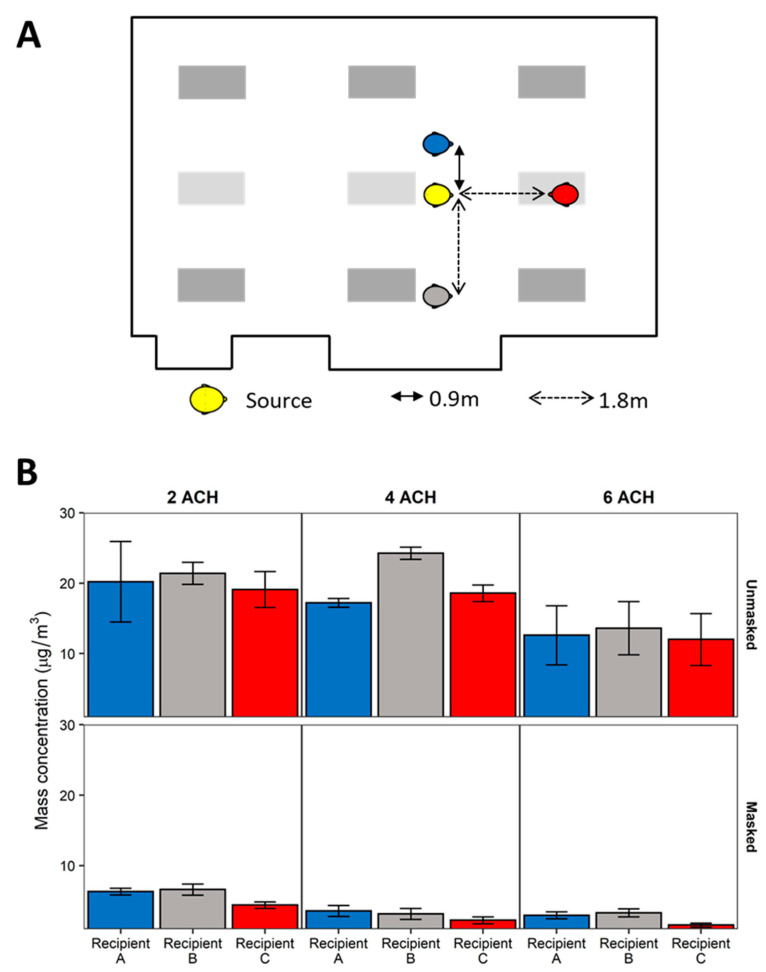
Effect of HVAC system ventilation. (**A**) Diagram of breathing simulator positioning for the experimental setup examining the effect of HVAC system ventilation. (**B**) Mean mass concentration measured at the Recipient breathing simulators for unmasked and universal masking experiments across the three HVAC system ventilation rates. Data are presented as the mean of four independent experiments. Error bars represent one standard deviation.

After assessing exposure reduction strategies, the positions of the breathing simulators were fixed in accordance with the guidance of maintaining minimally 0.9 m between simulators as demonstrated in Figure 2B. The change in aerosol exposure over the three HVAC ventilation rates (2, 4, and 6 ACH; Figure 4) was examined. When controlling for physical distance, orientation, and masking, increasing the HVAC system ventilation led to a 5.0% (SD 0.5%) decrease in exposure per unit increase in ACH (Supplemental Table S4). When stratified along masking, exposure reduction increased incrementally with each 1 ACH increase in HVAC ventilation when the breathing simulators were universally masked. When no masks were worn, exposure reduction among all Recipient breathing simulators was observed above 4 ACH. Universal masking significantly reduced exposure by 75.9% (SD 1.8%, *p* < 0.001). Physical distancing beyond 1.8 m did not statistically significantly influence exposure, while a side-by-side (vs. face-to-face) orientation relative to the Source significantly increased exposure (9.9% ± 2.2%, *p* < 0.001) compared to a face-to-face orientation which was pronounced under universal masking. Interpolation of relative particle concentrations reported by the area sampling OPCs across the exposure plane by inverse density weighting revealed the mean mass concentration was higher on the side of the meeting room nearest the Recipient B with the HVAC system set to 2 and 4 ACH (Supplemental Figure S1). When all breathing simulators were unmasked and the HVAC set to 6 ACH, the relative mean mass concentration was highest and largely homogeneous at the front half of the meeting room where all breathing simulators were positioned. When all breathing simulators were masked, the relative mean mass concentration was highest near Recipient B and at the back of the room.

### 3.4. HEPA Air Cleaners

**Figure 5:**
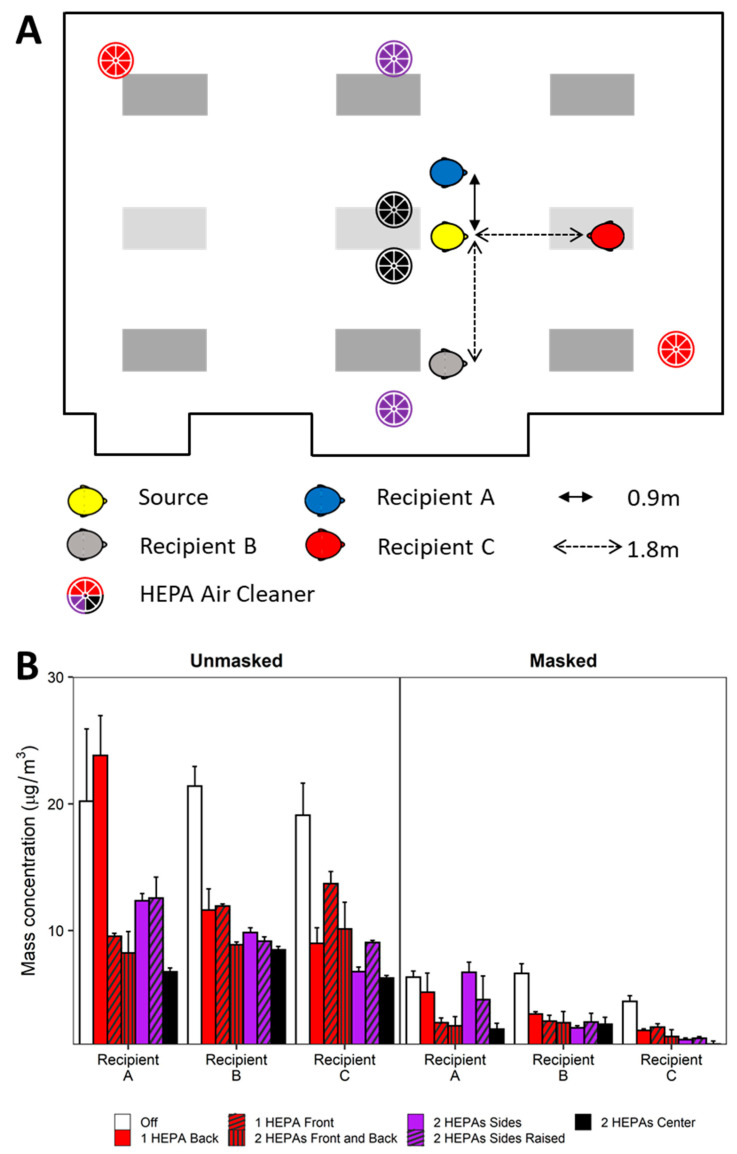
HEPA air cleaner augmentation. (**A**) Composite diagram showing breathing simulator in the second-row configuration and HEPA air cleaner placement scenario under HVAC system setpoint at 2 ACH. (**B**) Mean mass concentration measured at the Recipient breathing simulators for unmasked and universal masking experiments. Data are presented as the mean of four independent experiments. Error bars represent one standard deviation.

An examination was conducted to quantitate exposure reduction by augmentation of the HVAC system ventilation using one or two HEPA purifiers in various locations in the meeting room (Figure 5A). With an HVAC system ventilation of 2 ACH, the addition of either one or two HEPA air cleaners generally reduced exposure among the breathing simulator Recipients with notable exceptions (Figure 5B). Note that the combined flowrate produced using two HEPA air cleaners met the Environmental Protection Agency’s CADR recommendation, while using only one unit did not. When all breathing simulators were masked, the same HEPA configuration reduced Recipient A’s exposure to a lower extent compared to the other Recipients. Further, placement of two HEPA air cleaners at the sides of the meeting room (both on the floor and raised 0.3 m) reduced Recipient A’s exposure to a lower extent compared to the remaining Recipients. HEPA augmentation reduced exposure by 5.8% (SD 0.5%) per unit increase in ACH when controlling for masking, physical distance, and breathing simulator orientation (Supplemental Table S5). Similar to the HVAC system ventilation tests (Figure 4), universal masking significantly reduced exposure (*p* < 0.001) while physical distancing beyond 1.8 m had no effect. When comparing the percent exposure reduction per unit ACH, there was agreement between the exposure reduction coefficients irrespective of the source of ventilation. For example, exposure was reduced by 5.0% (SD 0.5%) per 1 ACH increase by the HVAC system compared to 5.8% (SD 0.5%) per 1 ACH increase provided by the HVAC system + HEPA augmentation.

**Figure 6:**
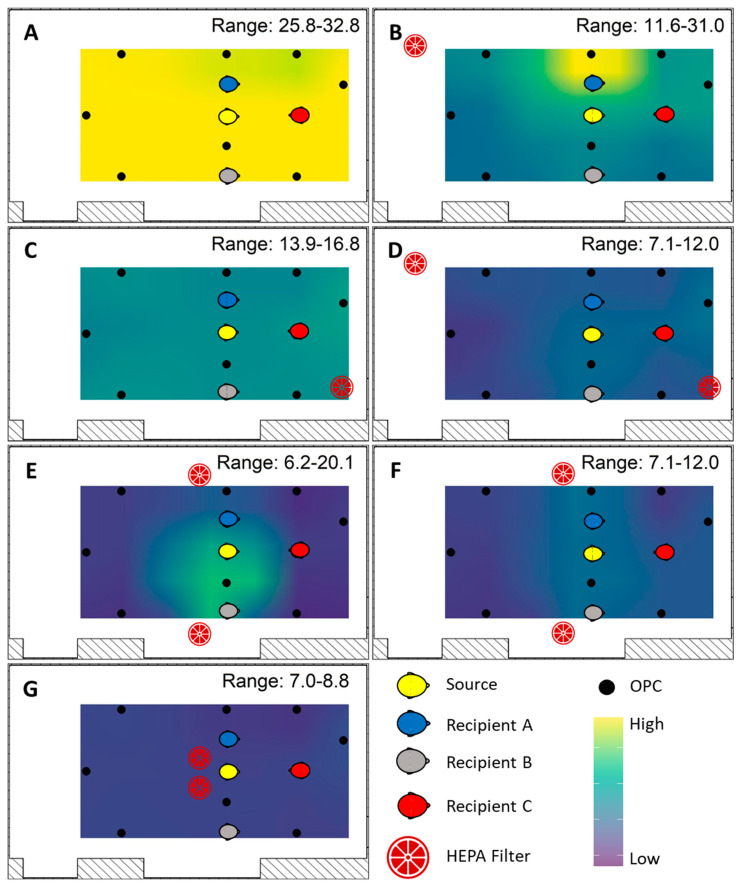
Spatial mean mass concentration distribution. The mean mass concentration of the area samplers was quantified and overlain on the HEPA configuration diagrams for the unmasked condition; analogous diagrams for the masked situation are included in Supplemental Figure S1. For each of the HVAC system ventilation and HEPA air cleaner configuration pairings, a separate geospatial map of the area samplers is presented: (**A**) HVAC system ventilation set at 2 ACH without HEPA augmentation; (**B**) one HEPA air cleaner placed in the back; (**C**) one HEPA air cleaner placed in the front; (**D**) two HEPA air cleaners placed in the front and back; (**E**) two HEPA air cleaners placed at the sides; (**F**) two HEPA air cleaners placed at the sides and raised upon 0.8 m tables; and (**G**) two HEPA air cleaners placed in the center of the room behind the aerosol source. Mean mass concentrations are the mean of four independent experiments. The coloration has been normalized to the concentration range observed among all trials, denoting purple as the lowest area sampler concentration and yellow as the highest. The relative spatial distributions of aerosols measured by area sampling were similar between unmasked and universal masking conditions, indicating that the room was well-mixed.

Differences among the individual Recipients within the examination of HEPA augmentation arise from the establishment of aerosol concentration pattern changes throughout the exposure plane (Figure 6). With the HVAC system ventilation at 2 ACH, the highest relative mean mass concentration was found at the back of the room nearest the side with Recipient B (Figure 6A). Deployment of a single HEPA air cleaner at the back of the room resulted in higher relative mean mass concentrations within the vicinity of Recipient A (Figure 6B), while a single unit at the front mixed the aerosol throughout the room (Figure 6C). Placement of two HEPA air cleaners, one at the front and one at the back of the room, also resulted in an aerosol mixture throughout the room. When HEPA air cleaners were placed at the sides of the room, the relative mean mass concentration was highest at the sides of the room nearest the Recipient simulators (Figure 6E,F). HEPA air cleaners placed at the center of the room directly behind the Source distributed the aerosol along the side positioned with Recipient B and at the back of the room (Figure 6G). When all breathing simulators were masked, the relative mean mass aerosol concentration patterns were largely similar, except the highest concentrations were measured at the back of the room when two HEPAs were used—one front and one back (Supplemental Figure S2). Nonetheless, the mean mass concentration measured was substantially lower than that in unmasked scenarios.

## 4. Discussion

Increases in HVAC ventilation, the use of HEPA air cleaners, universal masking, and physical distancing are all recommended interventions to reduce exposure to airborne particles carrying the SARS-CoV-2 virus. Many studies have looked at these interventions individually, but few have examined them in combination with each other. Assessments of the total effects of different combinations of ventilation, HEPA filtration, masking, and distancing are needed because these interventions can interact in ways that are not always obvious.

Increasing the HVAC system ventilation rate is an effective engineering control for reducing exposure to potentially infectious respiratory aerosols. For traditional HVAC systems, the highest removal rates generally occur when the air is well-mixed [44,45]. We observed reductions in the mass aerosol concentrations at the Recipients with increasing air exchange rates. These results are in line with the notion that released aerosols disperse more rapidly throughout the room at higher ACH rates because increases in ventilation provide more extensive air mixing in addition to increased aerosol clearance [46,47,48]. In addition to ventilation rates, room air currents can also affect exposure to aerosols and droplets, depending upon the position and orientation of a Recipient relative to the Source’s plume. This was especially notable for the Source within the participant area and the speaker when they were face-to-face. Since the relative aerosol concentration tended to be higher near Recipient B (a product of room airflow dynamics) and the speaker was directly in the path of the aerosol plume, the exposure for these two Recipients did not decrease until 6 ACH when no masks were worn (Figure 4).

Recognizing that the ventilation and airflow patterns observed are unique to the meeting room in which the experimentation was conducted, some generalizations can be drawn from the simulated scenario within the context of classroom-style room configurations, such as the meeting room simulation in the current investigation as well as classrooms. Examinations of air change rates in meeting rooms specifically are generally lacking, and measured ventilation rates for general office buildings and schools are generally under 2 ACH in the United States [24,49]. To address situations where simply increasing ventilation via the HVAC system is not possible, portable HEPA air cleaners offer a simple, relatively inexpensive option to increase indoor room ACH rates without modification to building infrastructure. Augmentation using either one or two HEPA air cleaners generally reduced Recipient mass aerosol concentrations by an additional 50% compared to 2 ACH by the HVAC ventilation alone. Overall, our results demonstrated that augmentation of the HVAC system with two HEPA air cleaners significantly decreased exposure, consistent with previous findings [27], though the position was an important factor in Recipient exposure. Mean mass concentrations were consistently lowest when two HEPA air cleaners were positioned with one unit in the front and one in the back of the room, both units raised on a table at the sides of the room, and both units in the center of the room. These three HEPA placements also dispersed aerosols homogeneously throughout the room, thus reducing the possibility of observing localized, high concentration areas as observed for the single HEPA air cleaner at the back of the room and two air cleaners placed on the floor at the sides of the room. The center placement, however, may not be feasible for all configurations and introduces considerations regarding space availability within the participant area for the HEPA air cleaners, noise, and power supply. 

Overall, HEPA deployment resulted in significant reduction in Recipient exposure, showing that the increase in total ACH provided by the HEPA air cleaners can reduce exposure to potentially infectious aerosols. However, it should be noted that the HEPA air cleaner units did not provide exposure reductions at all locations in all scenarios. Individual Recipient exposure metrics were dependent upon relative position of the Recipient to both the Source and the HEPA air cleaner(s). The addition of HEPA air cleaners will affect room air dynamics that, depending on Source and Recipient locations, could impact an individual Recipient’s exposures under certain scenarios. To reduce the likelihood of such an occurrence, HEPA positioning should be evaluated carefully to prevent the potential of drawing directed air currents from one occupant over another. Such an evaluation can be aided using handheld tracer “smoke” or “fog” generators. The use of multiple HEPA air cleaners spread out around the room provides a faster and better mixing and cleaning of the room air, thereby reducing the overall concentrations for participants in the room and reducing the probability of SARS-CoV-2 transmission.

For the scenarios with universal masking, the redirection of the exhalation airflow by the masks meant that the dispersion of the aerosol depended more upon the air currents induced by the combination of HVAC system and HEPA air cleaners and less on the air currents generated by breathing. This was readily observed when two HEPA air cleaners were placed at the sides of the room, wherein the HEPA air cleaners drew the aerosols towards the two Recipients adjacent to the Source. Here, Recipient A was positioned between the Source and the HEPA air cleaner while Recipient B was slightly to the side. This once again demonstrates the importance of evaluating HEPA placement to avoid unintended consequences. Nonetheless, overall, augmentation of the HVAC system with two HEPA air cleaners *in addition to* universal masking provided significant reductions in aerosol mean mass concentrations compared to no mitigation strategies.

Masking reduces aerosol release into the indoor environment during coughs and exhalations (called source control). Considerable research has focused on masks both as source control devices [13] and personal protective devices for a recipient [50,51]. When a mask is worn, exhaled aerosols may travel through the mask material and be partially or completely filtered out, or they may escape through gaps between the mask and the face (called face seal leaks) [52]. Aerosols that pass through or around the mask will disperse in the room and lead to changes in the relative aerosol concentration pattern. Previous work by our group has shown that with a face-to-face orientation at 1.8 m during breathing, universal masking reduced the mean aerosol exposure of a Recipient breathing simulator by 76% [39]. The results from the current investigation also showed similar aerosol mass concentration reduction by universal masking among the Recipients. 

To date, epidemiological studies have examined physical distancing effectiveness in schools, while those in office settings are lacking. Some schools, for example, had implemented recommendations of varying physical distance, thus setting up natural experiments for which the effect of physical distancing on class incidence rates may be examined. A retrospective cohort study comparing SAR-CoV-2 incidence rates between school districts adhering to ≥0.9 m and ≥1.8 m physical distancing during a 16-week study period reported no difference in incidence rate between the two districts [53]. Since that study, the CDC and the American Academy of Pediatrics have recommended maintaining a physical distance of ≥0.9 m between students, while physical distance recommendations between adults and students and between adults remain ≥1.8 m [54,55]. In our study, among the scenarios where the Source was a meeting participant, aerosol reduction by increasing physical distance was significant when controlling for orientation and masking (Figure 2). These results were likely influenced by the high exposures of the Recipient in the speaker position when the Source was placed at a distance of 0.9 m in a front-to-front orientation with the Recipient in the speaker position (Figure 2A) compared to 1.8 m (Figure 2B). While orientation itself was not an overall significant predictor of exposure, the combination of a front-to-front orientation and 0.9 m separation distance was the worst-case exposure scenario for a Recipient in the speaker position. Therefore, our results lend support to the ≥1.8 m physical distancing recommendations when the Source and a Recipient are oriented face-to-face. The results demonstrate that a complex interplay between physical distance, orientation, and room air currents exists. Within a close physical distance, i.e., 0.9 m, and without universal masking, a face-to-face orientation contributes significantly to the mean mass concentration exposure, as shown for the Recipient in the speaker position in Figure 2A compared to Recipient A that was side-by-side with the Source. However, when increasing physical distancing to 1.8 m or greater, the resulting face-to-face orientation exposure was similar to exposures observed for the two side-by-side Recipients. Thus, the interaction between orientation and physical distance should be understood within the context of exhalation plume volumetric dynamics. With increasing distance, the exhalation plume dissipates volumetrically, thus reducing the concentration of aerosols and subsequent exposure [36]. In the current investigation, we observed that the exhalation plume largely diluted the exposure of a face-to-face Recipient at 1.8 m and further to the general room mean mass aerosol concentration. For a Recipient with a side-by-side orientation to the Source, the air currents induced by the ventilation dominated the individual Recipient exposure, thus negating the effects of physical distancing for side-by-side orientations relative to the Source. Area monitoring revealed that with the ventilation ACH under 4.0, aerosols from the Source tended to accumulate on the side of the room where Recipient B was located, indicating that Recipients at any distance along this side of the experimental meeting room would have a higher exposure than comparable positions on the opposing side. In effect, for individuals in other than face-to-face orientations, the airflow patterns of the room may dictate the magnitude of exposure more readily than physical distancing alone. Since the room airflow patterns will be unique to the specific indoor environment, effective exposure mitigation by physical distancing greater than 0.9 m cannot be assured simply by modification of interpersonal distance alone.

Comparisons of the results in the current investigation to epidemiological findings must be made with caution. However, epidemiological findings examining the effects of masking, physical distancing, and/or ventilation generally support the findings from these simulations. For example, the relative risk of COVID-19 infections was significantly reduced among a cohort of elementary school students in Georgia by broad modifications of ventilation including dilution with fresh air and HEPA filtration, ultraviolet germicidal irradiation, and combinations thereof, as well as mandatory masking of teachers and staff. Physical distancing was not a significant determinant of case incidence [56]. These results agree with another examination of COVID-19 case incidence rates among public-school children in Massachusetts [53], though these results may be confounded through differential adherence to mask mandates and heterogeneities in ventilation among the schools [57]. Overall, our results support the general findings of the cohort studies demonstrating that masking and ventilation are strongly associated with aerosol reduction in the indoor environment and are reinforced by computational modeling [58,59]. While physical distancing may provide relatively minor reductions in exposure to very small aerosols, it still may provide an additional layer in a mitigation strategy.

There are several limitations to our investigation. First, our study was limited to airborne particles from 0.3 to 3 µm in diameter, which is a size range that includes bioaerosol particles that are small enough to remain airborne for an extended time but large enough to carry pathogens. However, humans produce aerosol particles across a broad size distribution [60,61], and particles and droplets larger than the size range in our experiments would behave differently, especially regarding the distancing variable. Second, the simulators used in the study were static and did not contain heating sources for either the air exhaled or the area around the breathing simulator to resemble body heat. More than one source as well as a moving index case throughout a room could change the aerosol dispersion pattern, leading to changes in exposures. The action of body movement has been shown to affect an individual’s personal exposure [62,63], which is a factor unaccounted for in the current investigation. Anthropogenic thermal air currents and movement can alter how aerosols are dispersed around the personal breathing zone and subsequently inhaled. Additionally, human-exhaled breath is also warm and humid which can affect how aerosols interact with the room airflow [37]. Third, the effect of physical distancing among individuals adjacent to the source in the current investigation remains difficult to separate from the influences of air currents induced by the HVAC system, the HEPA air purifiers, or both. In the most common configuration, we noted air currents of the HVAC caused increases in mean mass concentrations nearest Recipient B. Therefore, the effect of HVAC air currents confounded the effect of physical distancing on mean mass concentration in the experimental meeting room. Fourth, the current investigation was conducted in one classroom-style meeting room with a unique HVAC setup that cannot be generalized to other rooms. As such, the exposure reduction effect of ventilation and HEPA air cleaner placement would be expected to change, depending upon the room examined. Finally, the choice of HEPA air cleaners for a classroom-style meeting requires consideration of their noise level during operation [28]. In our study, the use of one or two HEPA air cleaners, in addition to the HVAC system, exceeded 60 dB which may, in certain environments, be too excessive. Fifth, a mitigation strategy not considered in this study was the use of windows to introduce outside air to the room for increased ventilation. It has been demonstrated that opening windows to create cross ventilation in buildings and rooms with poor ventilation from the building HVAC system can help reduce SARS-CoV-2 transmission [64]. The conference room setup used for this study was an interior room with no windows to the outside to allow natural ventilation into the room. Therefore, the effect of natural ventilation could not be examined.

## 5. Conclusions

Reducing the spread of COVID-19 requires reducing exposure of people to human-generated respiratory aerosols carrying the SARS-CoV-2 virus. Our results support the use of a combination of strategies to reduce the concentration of these infectious aerosols in indoor environments. Increases in ventilation rates, the use of HEPA air cleaners, the implementation of universal masking, and physical distancing can all play a role in decreasing the exposure of room occupants to airborne SARS-CoV-2. In this investigation, universal masking was the most consistent and efficacious mitigation strategy. Masking reduced exposure by 50% or more compared to no masking. Ventilation reduced exposure by approximately 5% per 1 ACH increase, irrespective of whether increasing ventilation was achieved by increasing system HVAC or by the use of portable HEPA air cleaners. The placement of portable HEPA air cleaners was a factor in determining a Recipient’s exposure reduction. Nonetheless, the most effective portable HEPA air cleaner configuration was the use of two units at opposing sides of the room, e.g., one at the front and one at the back or one each at the sides of the room, or two units in the center of the room. Increasing physical distance between the Source and Recipients beyond 0.9 m provided minimal exposure reduction when oriented side-by-side. However, maintaining a physical distance of 1.8 m or more between the Source and a Recipient in the face-to-face orientation was critical.

## Figures and Tables

**Table 1:** Effective air change rates by particle decay and noise.

HVAC ACH Setting	HEPA Configuration	ACH ± SD	Noise ± SD (dB)
2 Air Changes/Hour	None	1.89 ± 0.14	35 ± 0
	1 HEPA—Front	4.56 ± 0.01	61 ± 1
	1 HEPA—Back	4.31 ± 0.02	59 ± 1
	2 HEPAs—Front and Back	7.14 ± 0.11	63 ± 1
	2 HEPAs—Sides	7.03 ± 0.05	63 ± 1
	2 HEPAs—Sides Raised	6.74 ± 0.16	N.D.
	2 HEPAs—Center	6.94 ± 0.03	64 ± 1
4 Air Changes/Hour	None	3.45 ± 0.31	37
6 Air Changes/Hour	None	5.03 ± 0.17	38

Point estimates are the mean and one standard deviation of three independent measurements. N.D. = not determined. ACH = air changes/hour.

## Data Availability

Data are available from the authors upon reasonable request.

## Supplementary Materials

The following are available online at https://www.mdpi.com/article/10.3390/v13122536/s1, Figure S1: Spatial mean mass concentration distribution of system ventilation, Figure S2: Spatial mean mass concentration distribution with HEPA augmentation, Table S1: Comparative ACH measurement methods, Table S2: Regression coefficients for Figure 2, Table S3: Regression coefficients for Figure 3, Table S4: Regression coefficients for Figure 4, Table S5: Regression coefficients for Figure 5.
