# Supplementary material for: Efficacy of Ventilation, HEPA Air Cleaners, Universal Masking, and Physical Distancing for Reducing Exposure to Simulated Exhaled Aerosols in a Meeting Room
Source: Viruses. 2021 Dec 17;13(12):2536. doi: 10.3390/v13122536 (PMC8707272; doi:10.3390/v13122536)
Supplement: Supplementary file 1 [file viruses-13-02536-s001.zip › viruses-1463492-supplementary.pdf]

Efficacy of ventilation, HEPA air cleaners, universal masking, and physical distancing for reducing exposure to simulated exhaled aerosols in a meeting room

Jayme P. Coyle [1], Raymond C. Derk [1], William G. Lindsley [1,*], Francoise M. Blachere [1], Theresa Boots [1], Angela R. Lemons [1], Stephen B. Martin, Jr. [2], Kenneth R. Mead [3], Steve Fotta [1], Jeffrey S. Reynolds [1], Walter G. McKinney [1], Erik W. Sinsel [1], Donald H. Beezhold [1] and John D. Noti [1]

[1] Health Effects Laboratory Division, National Institute for Occupational Safety and Health, Morgantown, WV 26505; nti2@cdc.gov (J.P.C.); rhd8@cdc.gov (R.C.D.); wlindsley@cdc.gov (W.G.L.); czv3@cdc.gov (F.M.B.); oph6@cdc.gov (T.B.); wrw0@cdc.gov (A.R.L.); sff4@cdc.gov (S.F.); jsr0@cdc.gov (J.S.R.); wdm9@cdc.gov (W.G.M.); eur2@cdc.gov (E.W.S.); zec1@cdc.gov (D.H.B.); ivr2@cdc.gov (J.D.N.)

[2] Respiratory Health Division, National Institute for Occupational Safety and Health, Centers for Disease Control and Prevention, Morgantown, WV 26505; stm9@cdc.gov (S.B.M.)

[3] Division of Field Studies and Engineering, National Institute for Occupational Safety and Health, Cincinnati, OH 45226; kcm3@cdc.gov (K.R.M.)

* Correspondence: wlindsley@cdc.gov; Tel.: +1-304-285-6336

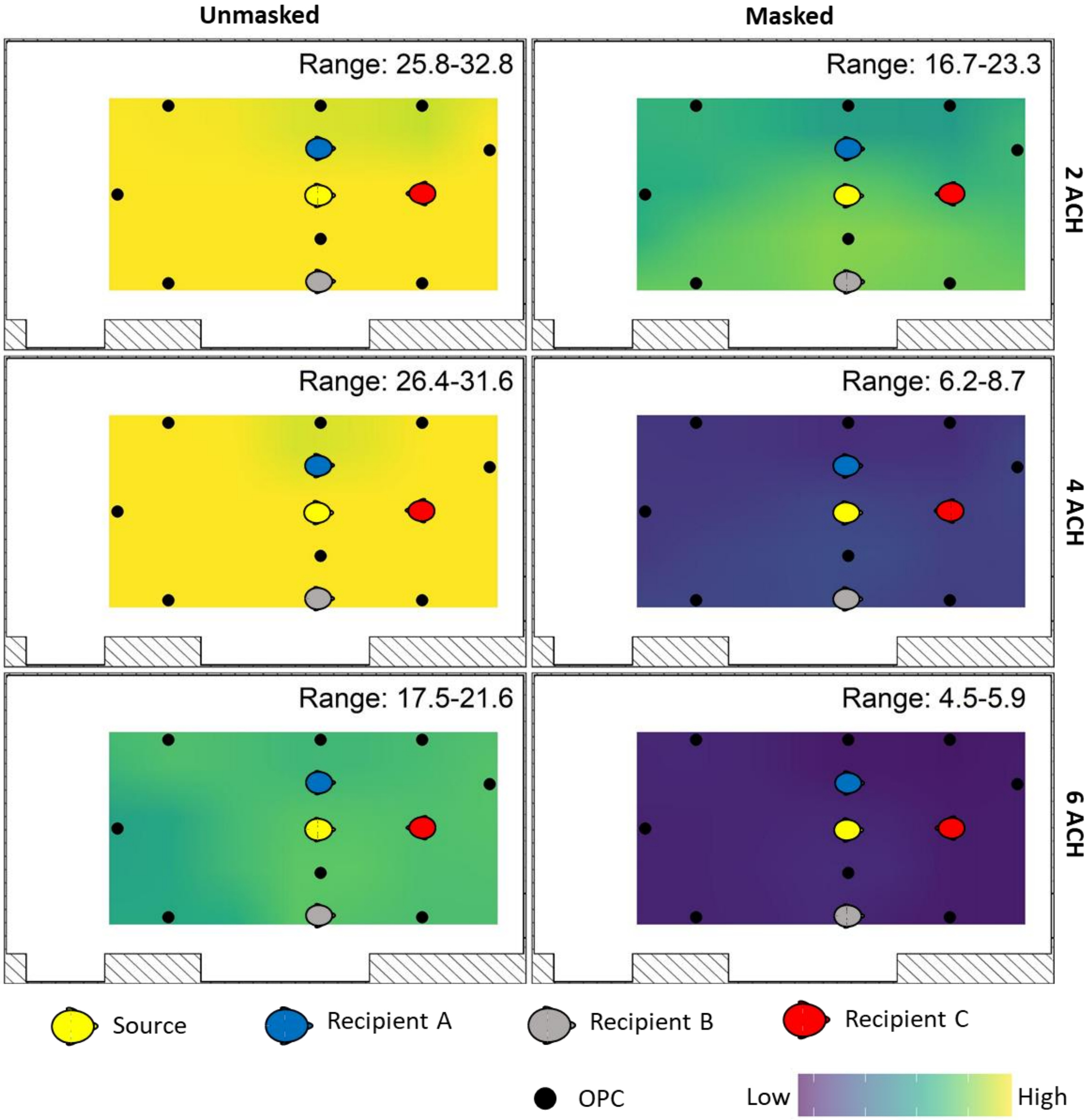

Figure S1. Spatial Mean Mass Concentration Distribution of System Ventilation. The mean mass concentration of the area samplers was quantified and overlain the matrix of HVAC system ventilation and masking diagrams. Mean mass concentrations are the mean of 4 independent experiments. Mean mass concentrations are the mean of four independent experiments. The coloration has been normalized to the concentration range observed among all trials, denoting purple as the lowest area sampler concentration and yellow being the highest.

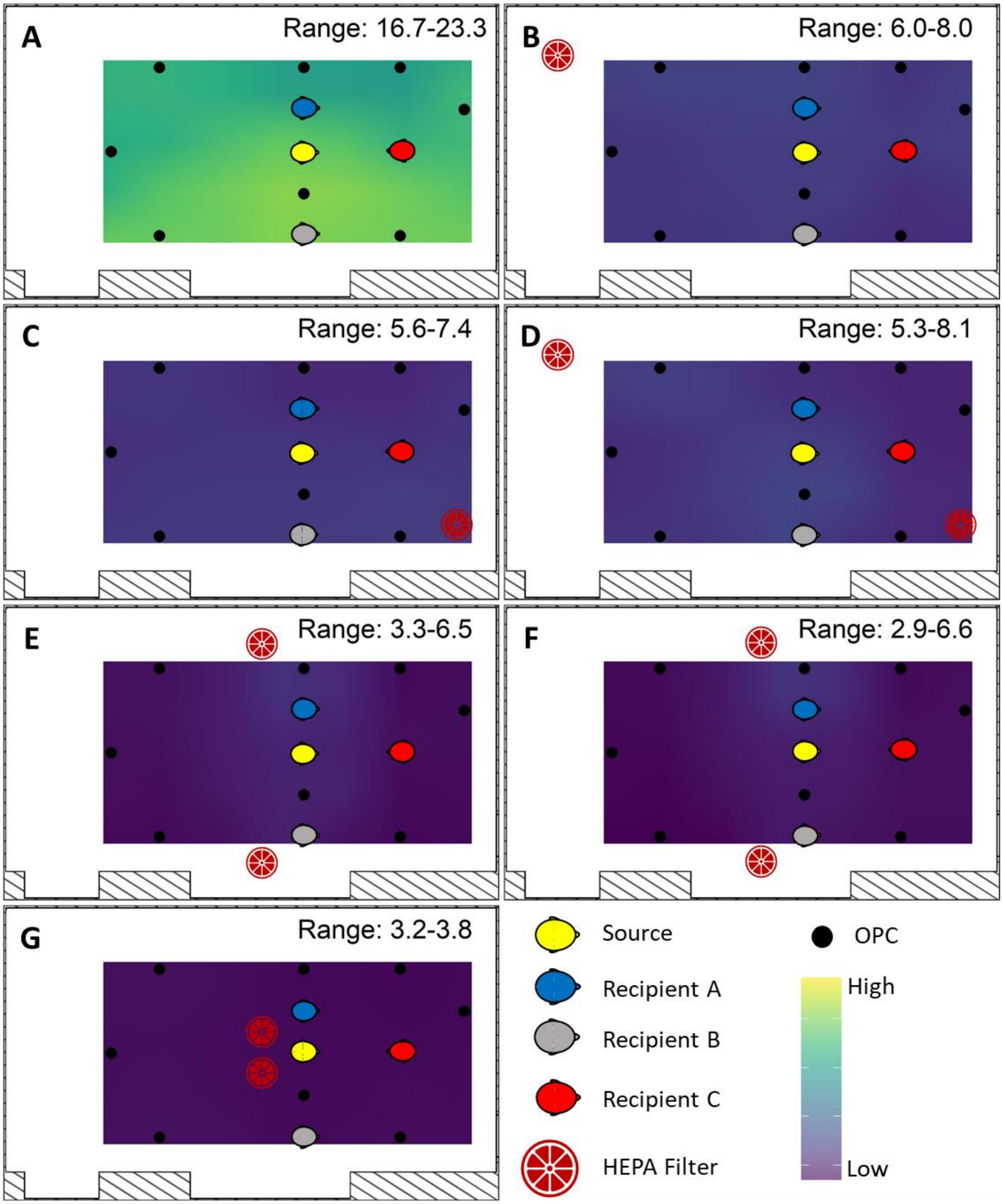

Figure S2. Spatial Mean Mass Concentration Distribution with HEPA Augmentation. The mean mass concentration of the area samplers was quantified and overlain on the HEPA configuration diagrams for the masked condition; analogous diagrams for the masked situation is included in

Supplementary Figure 1. For each of the system ventilation and HEPA air cleaner configuration pairing, a separate geospatial map of the area samplers is presented: (A) system ventilation set at 2 ACH without HEPA augmentation; (B) one HEPA air cleaner placed in the back; (C) one HEPA air cleaner placed in the front; (D) two HEPA air cleaners placed in the front and back; (E) two HEPA air cleaners placed at the sides; (F) two HEPA air cleaners placed at the sides and raised upon 0.8 m high tables; and (G) two HEPA air cleaners placed in the center of the room behind the aerosol Source simulator. Mean mass concentrations are the mean of 4 independent experiments. Mean mass concentrations are the mean of four independent experiments. The coloration has been normalized to the concentration range observed among all trials, denoting purple as the lowest area sampler concentration and yellow being the highest.

Table S1. Comparative ACH Measurement Methods

Measurement Method	2 ACH	4 ACH	6 ACH
Capture Hood*	2.09 ± 0.03	4.07 ± 0.03	6.08 ± 0.16
Tracer Gas	1.98 ± 0.02	3.68 ± 0.06	5.41 ± 0.03
Particle Decay	1.89 ± 0.14	3.45 ± 0.31	5.03 ± 0.17

Air exchange rates are presented as mean ± 1 SD of three individual experiments.

*Point estimate for an individual Capture Hood experiment was calculated as the room volume divided by the sum of individual readings taken from the 6 inflows to converted to air changes per hour (ACH).

Table S2. Regression Coefficients for Figure 2

Parameter	Coefficient	SE	CI95%	Exposure Reduction Percent	Exposure Reduction SE (Percent)
Intercept	17.99	1.56	-	-	-
Distance	-1.331	0.661	-2.643 to -0.018	-7.9%	3.9%
Universal Masking: Yes	-13.71	1.00	-15.69 to -11.73	-81.6%	5.9%
Orientation: Front-to-Front	0.660	1.604	-2.525 to 3.846	3.9%	9.5%
Orientation: Side-to-side	1.099	1.389	-1.659 to 3.857	6.5%	8.3%

Baseline parameters for Exposure Reduction is 0.9 m Distance, Unmasked, and Front-to-Back Orientation. SE denotes standard error derived from multiple linear regression model.

Table S3. Regression Coefficients for Figure 3

				Exposure Reduction	
**Parameter**	**Coefficient**	**SE**	**CI95%**	**Percent**	**SE (Percent)**
Intercept	15.28	0.57	-	-	-
Distance	-1.334	0.261	-1.881 to -0.787	-9.5%	1.9%
Universal Masking: Yes	-10.14	0.20	-10.55 to -9.73	-72.0%	1.4%

Comparison parameters for Exposure Reduction is 0.9 m Distance and Unmasked.

SE denotes standard error derived from multiple linear regression model.

Table S4. Regression Coefficients for Figure 4

Parameter	Coefficient	SE	CI95%	Exposure Reduction Percent	Exposure Reduction SE (Percent)
Intercept	18.49	0.90	-	-	-
Distance	0.659	0.420	-0.179 to 1.497	3.8%	2.4%
Universal Masking: Yes	-13.23	0.31	-13.84 to -12.61	-75.9%	1.8%
ACH	-0.879	0.095	-1.068 to -0.691	-5.0%	0.5%
Orientation: Side-to-side	1.723	0.378	0.969 to 2.477	9.9%	2.2%

Baseline parameters for Exposure Reduction is 0.9 m Distance, Unmasked, 1.89 ACH, and Front-to-Front Orientation. SE denotes standard error derived from multiple linear regression model. ACH = air changes per hour.

Table S5. Regression Coefficients for Figure 5

Parameter	Coefficient	SE	CI95%	Exposure Reduction Percent	Exposure Reduction SE (Percent)
Intercept	13.92	0.68	-	-	-
Distance	-0.466	0.307	-1.072 to 0.14	-3.8%	2.5%
Universal Masking: Yes	-7.193	0.226	-7.64 to -6.75	-59.1%	1.9%
ACH	-0.703	0.061	-0.823 to -0.583	-5.8%	0.5%
Orientation: Side-to-side	1.266	0.276	0.721 to 1.811	10.4%	2.3%

Baseline parameters for Exposure Reduction is 0.9 m Distance, Unmasked, 1.89 ACH, and Front-to-Front Orientation. SE denotes standard error derived from multiple linear regression model. ACH = air changes per hour.
